# 

**Published:** 1878-03

**Authors:** 


*CONTENTS.*

| | Page |
|---|---|
| Fever and Ague | 403 |
| Cholera Certainties | 408 |
| Reading | 412 |
| Wakefulness | 413 |
| The Family Relation | 414 |
| Married Life | 415 |
| Dollars and Ideas | 415 |
| Finger Nails | 416 |
| Cold Feet | 417 |
| Wanted Badly | 419 |
| Snuff Taking | 420 |
| School Children | 420 |
| Cookery | 421 |
| Lives of Medical Men | 421 |
| Death's Doings in England | 424 |
| How Life is Lost | 424 |
| Oderiferous Feet | 425 |
| A Queer Sight | 426 |
| Frost Work | 427 |
| Onions | 428 |
| Spirometer | 430 |
| Constipation | 431 |

